# Re.Ger.O.P.: An Integrated Project for the Recovery of Ancient and Rare Olive Germplasm

**DOI:** 10.3389/fpls.2020.00073

**Published:** 2020-02-20

**Authors:** Monica Marilena Miazzi, Valentina di Rienzo, Isabella Mascio, Cinzia Montemurro, Sara Sion, Wilma Sabetta, Gaetano Alessandro Vivaldi, Salvatore Camposeo, Francesco Caponio, Giacomo Squeo, Graziana Difonzo, Guiliana Loconsole, Giovanna Bottalico, Pasquale Venerito, Vito Montilon, Antonella Saponari, Giuseppe Altamura, Giovanni Mita, Alessandro Petrontino, Vincenzo Fucilli, Francesco Bozzo

**Affiliations:** ^1^Department of Soil, Plant and Food Sciences, University of Bari Aldo Moro, Bari, Italy; ^2^SINAGRI S.r.l.—Spin Off of the University of Bari Aldo Moro, Bari, Italy; ^3^Unit of Bari CNR Institute of Biosciences and Bioresources, Bari, Italy; ^4^Department of Agricultural and Environmental Sciences, University of Bari Aldo Moro, Bari, Italy; ^5^CRSFA-Centro Ricerca, Sperimentazione e Formazione in Agricoltura, “Basile Caramia” Locorotondo, Bari, Italy; ^6^Unit of Lecce, CNR Institute of Sciences of Food Production, Lecce, Italy

**Keywords:** olive, rare germplasm, homonymy and synonymy, characterization, diversity, resources for breeding

## Abstract

The olive tree is one of the most important economic, cultural, and environmental resources for Italy, in particular for the Apulian region, where it shows a wide diversity. The increasing attention to the continuous loss of plant genetic diversity due to social, economic and climatic changes, has favored a renewed interest in strategies aimed at the recovery and conservation of these genetic resources. In the frame of a project for the valorization of the olive Apulian biodiversity (Re.Ger.O.P. project), 177 minor genotypes were recovered in different territories of the region. They were submitted to morphological, molecular, technological and phytosanitary status analysis in comparison with reference cultivars, then they were propagated and transferred in an *ex situ* field. All the available information was stored in an internal regional database including photographic documentation and geographic position. The work allowed obtaining information about the genetic diversity of Apulian germplasm, to clarify cases of homonymy and synonymy, to check the sanitary status, and to identify candidate genotypes useful both to set up breeding programs and to enrich the panel of olive cultivars available to farmers for commercial exploitation.

## Introduction

The cultivated olive (*Olea europaea* subsp. europaea var. europaea) is a typical fruit tree crop of the Mediterranean Basin where it is spread on over eight million of hectares. In Italy, the olive culture represents one of the most important economic, cultural and environmental resource (Clodoveo et al., 2014; Famiani et al., 2014). The Italian olive germplasm is estimated to include about 800 cultivars, most of them landraces vegetatively propagated at a farm level since ancient times (Muzzalupo, 2012), and new local genotypes are continuously described. This wealth is due to the high environmental variability of Italian growing area, and it represents an opportunity for the Italian olive oil sector. The increasing attention to the continuous loss of plant genetic diversity, known as genetic erosion, due to social, economic and climatic changes, determined targeted international policies to preserve plant species subjected to extinction risk. The International Treaty on Plant Genetic Resources for Food and Agriculture (FAO, 2001) created a mechanism for an equitable use of these resources and envisaged the creation of a global information system to facilitate the recovery and sharing of plant genetic resources (Sardaro et al., 2018). Plant biodiversity is a resource of genes useful to adaptation to environmental changes. The characterization of ancient and rare plant genetic resources is prominent as source of agronomical traits important for cropping system evolution (Caruso et al., 2014; Vivaldi et al., 2015; Rosati et al., 2018a; Rosati et al., 2018b), reduction of water consumption (Pellegrini et al., 2016), facing emergent diseases resistance (Saponari et al., 2019) and resilience to climate changes (Taranto et al., 2018).

Apulia region (Italy) hosts one third of the Italian olive cultivated area, with about 50 million of olive trees showing a wide diversity. The particular conformation of the Regional territory, stretched on more than 400 Km, offers a great variability of pedoclimatic conditions (Sardaro et al., 2015). Due to its geographical position, Apulia was a cross point for commercial routes since ancient times, allowing a remarkable complexity and richness in autochthonous varieties as documented in several researches (D'Agostino et al., 2018).

In 2013, the integrated project Re.Ger.O.P. (Apulian Olive Germplasm Recovery) focused the attention on the Apulian olive biodiversity through a structured program of activities including historical investigation, cataloguing, genetic and technological characterization, sanitary status investigation and conservation of the collected local germplasm.

Morphological descriptors are an important tool to study the genetic diversity within a cultivated plant species; indeed, they represent the phenological traits normally used in taxonomic classification (Blazakis et al., 2017). The International Union for the Protection of New Varieties of Plants (UPOV) established both parameters and methodology for olive germplasm characterization (UPOV, 2011).

Nonetheless, in the last two decades, the morphological descriptors have been integrated with molecular markers, such as SSR markers that have demonstrated a very good efficiency in olive genotyping (Muzzalupo et al., 2008; Muzzalupo et al., 2014; Boucheffa et al., 2017; Chiappetta et al., 2017; Boucheffa et al., 2019; Sion et al., 2019), population genetics (Albertini et al., 2011; Mousavi et al., 2017; di Rienzo et al., 2018a), and traceability of products (Pasqualone et al., 2015; Montemurro et al., 2015; Binetti et al., 2017; Sabetta et al., 2017).

An essential prerequisite in the genetic resources' conservation is also the assessment of the phytosanitary status (Fontana et al., 2019). As for other vegetative propagated crops, olive is affected by several pathogens (viruses, fungi, bacteria and phytoplasmas) that persist in the budwood and can be transmitted and disseminated with it, with severe economic effects on yield and production quality (Martelli et al., 2002; Loconsole et al., 2010). The use of ‘healthy’ plants for new plantations is crucial for the quality of crop production, restraining the spread of pathogens and diseases, and potentially reducing chemical applications and the environmental impacts of agricultural practices (García-Mier et al., 2013). Moreover, the use of local varieties, recovered from autochthonous germplasm, could support programs of resistance evaluation to emergent pests (Giampetruzzi et al., 2016; Saponari et al., 2019).

Despite the economic importance of olive (ISMEA, 2019), few major cultivars are generally used for virgin olive oil (VOO) production, neglecting the heritage of minor cultivars that could be an important resource to broaden the product offer to the consumers. Indeed, the genotype component, coupled with the extraction technology, strongly affect the VOO characteristics in terms of quality, oxidative stability and organoleptic features (Rotondi et al., 2010; Caponio et al., 2018a; Caponio et al., 2018b; Tamborrino et al., 2019).

The aim of this work was to obtain information about genetic variability among Apulian germplasm, to clarify cases of homonymy and synonymy, to check the sanitary status, and to identify candidate genotypes useful both to set up breeding programs and to enrich the existent panel of olive cultivars. The integrated approach here proposed allowed to reach a deep knowledge on several aspects connected to the olive germplasm for a faster and efficient recognition of the best candidates suitable for commercial and economic valorization.

## Materials and Methods

### Collection of Olive Germplasm

**Figure 1:**
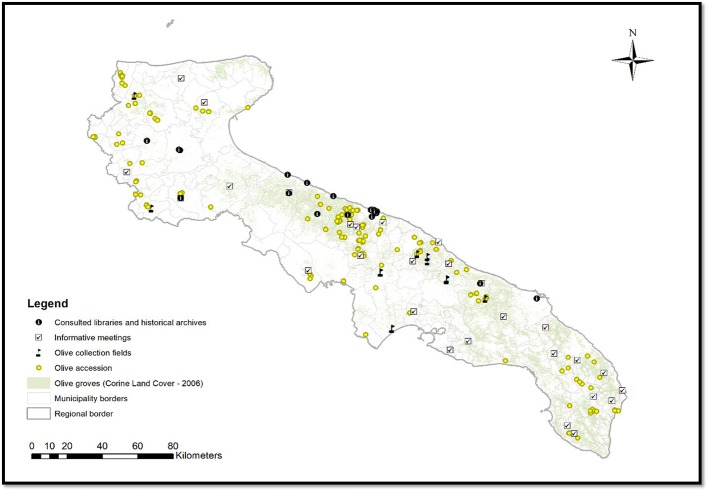
Geographic map of ‘Apulia’ with collection sites of samples.

To identify the minor genotypes spread in Apulia region (Southern Italy), bibliographic researches about the varieties cultivated in the past centuries were conducted in cooperation with local farmers and by means of meetings organized along the regional territory. Plants were recovered in the marginal areas of the Apulian provinces of Foggia, Bari, Brindisi, Taranto, BAT (Barletta–Andria–Trani) and Lecce (**Figure 1**). Genotypes were geo-referenced trough cartography and GPS data of the fields. In addition, a photographic documentation with a geotag system and 3D photographs for remote recognition of tree canopy, rural landscape and soil characteristics were obtained. A total of 177 genotypes were collected and they were submitted to the different characterizations, depending on the availability of the plant material (**Supplementary Table 1**).

### Morphological Characterization

The morphological characterization was performed on 97 genotypes using 24 descriptors indicated by UPOV, including three descriptors for the leaf, 11 for the fruit, and 10 for the stone (**Supplementary Table 2**). Each genotype was represented by one to three trees, depending on genotype; in few cases the tree consisted in a primary branch only. Observations were made on 50 leaves, 50 fruits and 50 stones per genotype. Leaves were collected in late spring, on 1-year shoots; fruits were harvested in middle autumn, with a pigmentation index between 3 and 5 (Camposeo et al., 2013). The morphological data were converted in a discrete data matrix (**Supplementary Table 3**) and used to obtain a Neighbour–Joining dendrogram performed with Darwin software version 6.0.010 (http://darwin.cirad.fr) (Felsenstein, 1985), using 10,000 bootstrap replications. The data were compared with the reference cultivars present in the OLEA database (OLEADB, http://www.oleadb.it) which includes 1,626 cultivars preserved in 102 field collections of different countries (Bartolini et al., 2014).

### Genetic Characterization

The molecular characterization was performed on 177 Apulian genotypes using 11 preselected microsatellite markers suitable for olive cultivar discrimination (Sefc et al., 2000; Carriero et al., 2002; Cipriani et al., 2002; Dela Rosa et al., 2002; Baldoni et al., 2009) (**Supplementary Table 4**). The obtained genetic profiles were compared with that of 59 olive cultivars diffused in all the Italian territory and maintained at the conservation field of Palagiano (TA, Italy), used as references (**Supplementary Table 1**). For genomic DNA extraction, three young leaves of each sample were lyophilized and finely grinded, and 50 mg of tissue were used, following the protocol of Spadoni et al. (2019). DNA quality and concentration were assessed using a NanoDropTM ND2000C (Thermo Fisher Scientific, Waltham, MA, USA), and normalized at 50 ng/μl into a 96-well plate (Nunc 96-Well Multiwell Plates). DNA amplification and analysis were conducted according to di Rienzo et al. (2018a). Detection, sizing and data collection were carried out using the GeneMapper 5.0 software (Applied Biosystems, Foster City, CA, USA).

Genetic diversity was investigated through different genetic indices, such as Number of alleles (Na), effective alleles (Ne), Shannon's information index (I), observed (Ho) and expected (He) heterozygosity and fixation index (F) (Wright, 1949) implemented in GENALEX software v.6.5 (http://anu.edu.au./BoZo/GenAIEx). This software was also used to calculate the allelic similarity for codominant data based on the pairwise relatedness, following the Lynch and Ritland estimator (LRM) (Lynch and Ritland, 1999). To determine the most informative primers, the polymorphic information content (PIC) (Botstein et al., 1980) was calculated by using Cervus v 3.0 (Kalinowski et al., 2007).

To study the relationships among genotypes, an Unweighted Neighbor–Joining dendrogram was generated in DARWIN software v. 6.0.010 (http://darwin.cirad.fr), using bootstrapping with 1,000 replicates to determine support for each node.

To infer the structure of olive germplasm, a Bayesian clustering algorithm implemented in STRUCTURE software version 2.3.4 (https://web.stanford.edu/group/pritchardlab/structure.html) (Pritchard et al., 2000) was used. To evaluate the optimal number of sub-populations (K), ten independent runs for each K (from 1 to 10) were performed, using 100,000 MCMC repetitions and 100,000 burn-in periods. The optimal K value was determined depending on ΔK test (Evanno et al., 2005) using the STRUCTURE HARVESTER software (Earl and Von Holdt, 2012). Genotypes were assigned to defined populations if the value of the corresponding membership coefficient (qi) was higher than 0.6, otherwise they were considered to be of admixed ancestry.

### Technological Characterization of Virgin Olive Oils

The technological characterization was performed on 34 genotypes (**Supplementary Table 1**) using 1 kg of olives from a homogeneous batch collected by hand during the harvest season 2014–2015 from at least two different trees, when the pigmentation index was about 2 (Squeo et al., 2016). The extraction of the corresponding monovarietal virgin olive oils (VOO) was obtained within 12 h after harvesting, using a semi-industrial scale hammer crusher (RETSCH GmbH 5657, Haan, Germania) provided with three hammers positioned at 120° on a single plane. Thirty counter beaters (height = 5 mm) were embedded in the side of the chamber with an angle of 42° while the lower part of the chamber was covered by a grid with 65 holes (φ = 5 mm). The angular velocity was set at 2,850 rpm (Caponio and Catalano, 2001). The recovered olive paste was indirectly heated at 30 ± 1°C using a hot water treatment and mixed for 15 min. Thereafter, the oily phase was collected by a basket centrifuge (Marelli Motori S.p.A., Arzignano, VI, Italia) with a bowl of 19 cm, at rotational speed of 2,700 rpm. Once extracted, the oils were stored in 100 ml dark glass bottles until the analyses.

For the technological characterization, fatty acids (FA) and sterols composition was determined as described respectively in Difonzo et al. (2018) and the Commission Regulation (EEC) No 2568/91. Briefly, for FA analysis, about 20 mg of oil was added with 1 ml of hexane and vortexed. Then, 1 ml of KOH solution in methanol (2 N) was added and the sample sonicated by an ultrasound bath (CEIA, Viciomaggio, Italy) for 6 min at 25°C. Two microliters of the recovered upper layer, containing the fatty acids methyl esters (FAME), were withdrawn and injected into the GC system (Regulation (ECC) No 2568/91). The GC-FID system was composed by an Agilent 7890A gas chromatograph (Agilent Technologies, Santa Clara, CA, USA) equipped with a FID detector (set at 220°C) and a SP2340 capillary column, 60 m × 0.25 mm (i.d.) × 0.2 μm film thickness (Supelco Park, Bellefonte, PA, USA). The identification of each fatty acid was carried out by comparing the retention time with that of the corresponding standard methyl ester (Sigma-Aldrich, St. Louis, MO, USA) and the results were expressed as area percentage respect to the total FAs area.

For sterols composition, about 5 g of oil were added with α-colestanol as internal standard, and sample was subjected to saponification with a solution of KOH in ethanol (2 N) under heating. Sample was transferred in a separating funnel and washed three times with ethyl ether in order to collect the unsaponifiable fraction. The etheric phase was neutralized and filtered by sodium sulphate anhydrous and dried. The sterol fraction, resuspended in chloroform (5%), was separated from the unsaponifiable matter by tin layer chromatography and then recovered, filtered and silanised. Finally, about 1 μl of the solution was injected in the GC system (Agilent 7890A) using a capillary column HP-5 30 m × 0.32 mm (i.d.) × 0.25 μm film thickness (Agilent Technologies, Santa Clara, CA, USA). The injector temperature was 290°C with a split ratio of 1:25. The identification was carried out by comparing the retention time with those reported in the official method (Commission Regulation (EEC) No 2568/91). Single sterols content was reported as area percentage respect to the total sterol area, while the total content was calculated using the internal standard method and expressed as mg kg. All the chemical analyses on VOOs were made in triplicate, in all cases, with a coefficient of variation <5%. Descriptive statistics of the VOOs characteristics were calculated using Microsoft Excel 2010 (Microsoft Inc., Redmond, WA, USA). The principal component analysis (PCA) was also performed and the CAT (Chemometric Agile Tool) R-based chemometric software (R version 3.1.0 (2014-04-10) on the autoscaled matrix, was used.

### Phytosanitary Evaluation of Olive Germplasm

The phytosanitary characterization was performed on 129 accessions, assessing either the presence of viruses listed in the phytosanitary requirements (D.M 20/11/2006) (**Supplementary Table 1**; **Supplementary Table 5**) and the bacterium *Xylella fastidiosa*. For the viruses, a previously validated one tube RT-PCR protocol was used (Loconsole et al., 2010), while for *X. fastidiosa*, the standard procedure based on CTAB-protocol for the extraction of total DNA and qPCR was applied (Harper et al., 2010; EPPO - PM 7/24 (3), 2018). Olive accessions resulted infected by CLRV and OLYaV, were submitted to sanitation treatments by *in vivo* or *in vitro* thermotherapy (Bottalico et al., 2004; Acquadro et al., 2009; Chiumenti et al., 2013; Abou Kubaa et al., 2018; Spanò et al., 2018). In *in vivo* thermotherapy, 2-year old infected plants were exposed to 35–38°C for 3–4 months; successively their vegetative tips were excised and micro-grafted on 1-year old “virus-free” seedlings (Onay et al., 2004; Wu et al., 2007; Farahani et al., 2011) (**Supplementary Figure 1**). In *in vitro* thermotherapy, young shoots were excised from 2-year old infected plants; nodal cutting explants were surface sterilized washing in running water and dipping in a NaClO (7–9% Cl active) solution for 20 min, then washed in steril water for three times for 1'–2' for each one. The explants were cultivated in Petri dishes with 25 ml of Olive media (Rugini, 1984) modified with zeatin 1.0 mg l^−1^ and mannitol 36 g l^−1^. Petri dishes were maintained in growth chamber at 24°C with a 16 h light/8 h dark period and 3,000 lx light intensity. The *in vitro* sprout shoots were subcultivated on the same media composition every 20 days for 4 months. When shoots became about 2 cm long they were submitted to *in vitro* thermotherapy. The shoots transferred in glass vessels with 100 ml of the same media were exposed to 35–38°C, 16 h light/8 h dark period and 3,000 lx light intensity for 1 month, then their vegetative tips (0.5–0.8 cm) were directly micro-grafted on “virus-free” seedlings. The micrografted plants were protected by a plastic bag and maintained in a chamber room for 1 month at the same condition described before. Plastic bags were gradually removed, and the plants were transferred in greenhouse. Micrografted plants resulted negative to OLYaV and CLRV at a first testing, were maintained in greenhouse and checked two times in 18–24 months after the first assay. Finally, ‘healthy plants’ were transferred in screen house and maintained isolated to avoid any type of contamination, and routinely tested for the pathogens reported before.

## Results

### Plant Material

Historical investigations about the varieties locally cultivated in the past centuries in Apulia, and the prospections on the territory allowed to identify a total of 177 minor genotypes survived in marginal olive orchards (**Supplementary Table 1**; **Figure 1**). All the genotypes were geo-referenced through cartography and GPS, and all data were merged in a database specifically created as repository of molecular, morphological, phytosanitary and technological information (data available on request to Apulia Region Misura 10.2.1: www.psr.regione.puglia.it).

### Morphological Characterization

**Figure 2:**
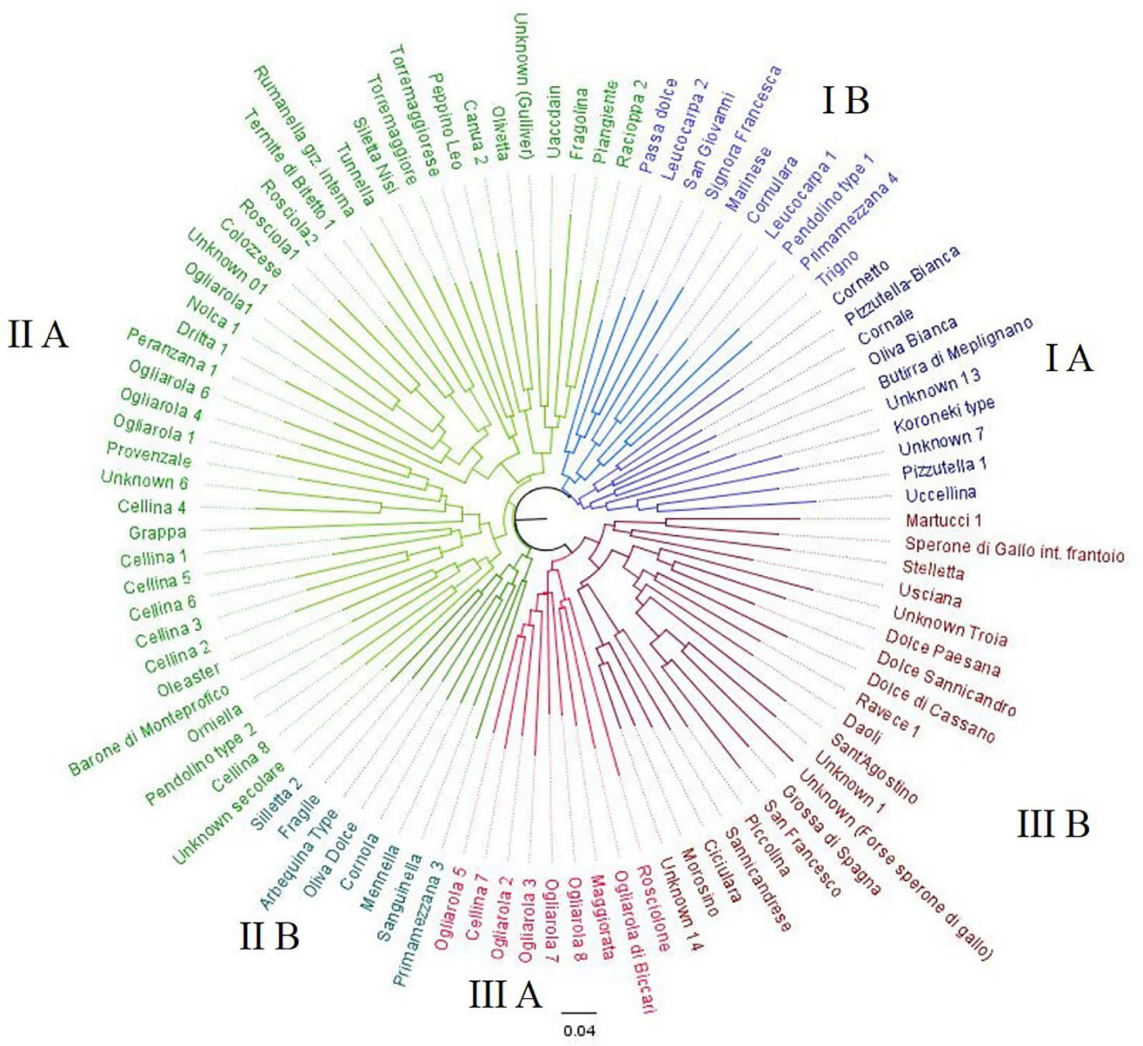
Dendrogram generated by Neighbor-Joining clustering method, illustrating the relationships among 97 olive Apulian genotypes, using the morphological markers.

Fruit and stone separated the 97 Apulian olive genotypes in clusters and allowed to recognize 32 varieties already described in OLEADB, as well-known cultivated varieties. Among the remaining 64 genotypes, 49 genotypes were not described in OLEADB but they were called with a local name, and 15 genotypes were nameless and they were tagged as “unknown”. The dendrogram grouped the olive genotypes in three main clusters (**Figure 2**). Cluster I consists of 20 genotypes including the white-fruit phenotype such as ‘Oliva bianca’ (I A) and ‘Leucocarpa 1’ (I B), and all the genotypes characterized by pointed apex and asymmetric stone, such as ‘Cornale’, ‘Cornulara’ and ‘Pizzutella 1’. This cluster also includes four unknown genotypes (Unknown-7, Unknown_13, Pendolino-type 1, Koroneiki-type) and eight not described genotypes: ‘Cornetto’, ‘Leucocarpa 2’, ‘Passa dolce’ ‘Pizzutella bianca’, ‘Primamezzana 4’, ‘San Giovanni’, ‘Signora Francesca’, and ‘Trigno’. Cluster II included 48 genotypes, 25 not present in OLEADB (‘Canua 1’, ‘Fragile’, ‘Fragolina’, ‘Grappa’, ‘Mennella’, ‘Orniella’, ‘Primamezzana 3’, ‘Rumanella’, ‘Sanguinella’, ‘Silletta 2’, ‘Silletta Nisi’, ‘Torremaggiorese’, ‘Torremaggiore’, ‘Uaccdain’, four ‘Ogliarola’, seven ‘Cellina’), and one oleaster. Cluster III comprises 29 genotypes characterized by medium-high fruit size and stone weight and size: three genotypes not described in OLEADB (‘Ogliarola di Biccari’, ‘Oliva maggiorata’ and ‘Rosciolone’ etc.), five accessions of ‘Ogliarola’ and ‘Cellina 7’ (subcluster IIIA), three table olives cultivars (‘Grossa di Spagna’, ‘Sant'Agostino’ and ‘San Francesco’), and five other genotypes (Unknown-1, Unknown-14, ‘Troia’, ‘Sperone di Gallo’ and ‘Martucci 1’) (sub-cluster III B). This sub-cluster also includes two of the most typical ‘sweet olives’, ‘Dolce di Cassano’, and ‘Dolce di Sannicandro’, and seven local genotypes (‘Ciciulara’, ‘Daoli’, ‘Dolce paesana’, ‘Morosino’, ‘Piccolina’, ‘Sannicandrese’, and ‘Stelletta’). Most of “unknown” genotypes showed high similarity with cultivars ‘Ogliarola’ and ‘Cellina’; only ‘Unknown 3’ and ‘Unknown 5’ did not revealed similarity with any other variety, showing a unique profile (**Table 1**).

**Table 1:** Results of the clustering of the 97 Apulian genotypes based on morphological traits. Known genotypes indicate genotypes present in the databank OLEADB.

Cluster	Sub-cluster	Profile	Genotypes described in OLEADB	Genotypes undescribed in OLEADB	Unknown genotypes	Other new genotypes
I	I A	1	Pizzutella, Uccellina	–	Unknown 7, Koroneiki-type	–
		2	Butirra di Melpignano	–	Unknown 13	–
		3	Cornale, Marinese, Oliva bianca	Cornetto, Pizzutella bianca	–	–
	I B	4	Leucocarpa 1, Cornulara	Trigno, Primamezzana 4	Pendolino-type 1	–
		5	–	Leucocarpa 2, Passa dolce, San Giovanni, Signora Francesca	–	–
II	II A	6	Piangente, Racioppa	Fragolina, Uaccdain	Unknown (Gulliver)	–
		7	Peppino Leo, Olivetta	Canua 1,	–	–
		8	–	Silletta Nisi, Torremaggiore Sacco, Torremaggiorese	–	–
		9	Colozzese, Dritta, Nolca, Rosciola (1, 2), Termite di Bitetto, Tunnella	Rumanella, Ogliarola 1	Unknown 01	–
		10	Peranzana	–	–	–
		11	Provenzale	Grappa, Cellina 3, Ogliarola 2,5,7,	Unknown 6	–
		12	–	Cellina 2,4,5,8,9		–
		13	Barone di Monteprofico	Orniella, Cellina 6	Pendolino-type 2	Oleaster
		14	–	–	Unknown secolare	–
	II B	15	Corniola, Oliva dolce	Fragile, Mennella, Silletta	Arbequina-type	–
		16	–	Primamezzana 1, Sanguinella		–
III	III A	17	–	Ogliarola 3,4,6,8,9, Cellina 7		–
		18	–	Ogliarola di Biccari, Oliva maggiorata, Rosciolone	–	–
	III B	19	–	Ciciulara, Morosino, Piccolina, Sannicandrese,	Unknown 14	–
		20	Grossa di Spagna, Sant'Agostino, San Francesco	Daoli	Unknown 1, Unknown (SG)	–
		21	Dolce di Cassano, Ravece	–	–	–
		22	Dolce di Sannicandro	Dolce paesana	Unknown Troia	–
		23	Sperone di gallo, Usciana	Stelletta	Martucci 1	–

### Genetic Characterization

The eleven selected SSR markers successfully amplified the 177 olive studied samples, confirming to be highly informative as indicated by PIC values that was higher than 0.5 at all loci (mean 0.72), except for EMOL (**Table 2**). Total number of alleles (Na) of 113 alleles was obtained, with a mean of 10 alleles per locus, ranging from four alleles for EMOL to 17 alleles for DCA09. The average number of effective alleles (Ne) was 4.86, ranging from 1.57 (EMOL) to 11.02 (DCA09). The observed Heterozygosity (Ho) varied between 0.10 for EMOL and 0.89 for DCA09 (mean Ho = 0.64), whereas the expected Heterozygosity (He) ranged between 0.36 (EMOL) and 0.91 (DCA09) (mean He = 0.74). The Fixation Index (F) was positive for all markers except for DCA05, GAPU71b and GAPU101. The Na in the reference cultivars was higher (143), and Ho (mean 0.71) was lower than He (mean 0.79). On the whole collection (local genotypes and reference cultivars), the total Number of alleles was 172 (**Table 2**), with a mean of 16 alleles per locus, ranging from 10 alleles for EMOL to 27 alleles for DCA18. The average number of effective alleles (Ne) was 6.43, ranging from 1.79 (EMOL) to 13.61 (DCA09). Shannon's information index (I) ranged from 0.93 (EMOL) to 2.73 (DCA09). The observed Heterozygosity (Ho) varied between 0.19 for EMOL and 0.85 for GAPU101 (mean Ho = 0.66), whereas the expected Heterozygosity (He) ranged between 0.44 (EMOL) and 0.93 (DCA09) (mean He = 0.80). The Fixation Index (F) was positive for all markers except for GAPU71b. The genetic analysis was also carried out on the three clusters resulting by the Neighbor Joining Analysis (**Supplementary Table 6**). The Na for the cluster I (11 genotypes), the cluster II (87 genotypes) and the cluster III (137 genotypes) was 70, 162 and 106, respectively, while Ne was 4.23, 7.53 and 4.68, respectively.

**Table 2:** Genetic indices obtained by SSR analysis on 177 minor Apulian accessions.

Minor Apulian genotypes (177)	Locus	Na	Ne	I	Ho	He	F	PIC
	**DCA03**	11	6.48	2.04	0.82	0.85	0.03	0.829
	**DCA05**	11	3.82	1.74	0.76	0.74	−0.02	0.716
	**DCA09**	17	11.02	2.51	0.89	0.91	0.03	0.902
	**DCA13**	8	3.24	1.55	0.44	0.69	0.37	0.664
	**DCA15**	9	3.37	1.49	0.35	0.70	0.50	0.662
	**DCA17**	15	4.99	1.94	0.65	0.80	0.19	0.777
	**DCA18**	14	6.52	2.13	0.77	0.85	0.09	0.83
	**GAPU71b**	8	3.99	1.54	0.83	0.75	−0.11	0.713
	**GAPU101**	8	5.04	1.76	0.85	0.80	−0.07	0.773
	**EMO90**	8	3.44	1.47	0.56	0.71	0.20	0.67
	**EMOL**	4	1.57	0.68	0.10	0.36	0.72	0.332
	**TOTAL**	113						
	**Mean**	10	4.86	1.71	0.64	0.74	0.18	0.72
Reference cultivars (59)								
	**DCA03**	11	5.22	1.92	0.88	0.81	−0.09	0.79
	**DCA05**	11	3.91	1.74	0.73	0.74	0.02	0.72
	**DCA09**	16	10.45	2.50	0.69	0.90	0.24	0.90
	**DCA13**	14	3.22	1.69	0.51	0.69	0.26	0.67
	**DCA15**	7	3,61	1.53	0.73	0.72	−0.01	0.69
	**DCA17**	17	6.88	2.25	0.62	0.85	0.27	0.84
	**DCA18**	22	9.02	2.56	0.76	0.89	0.14	0.88
	**GAPU71b**	10	4.97	1.89	0.84	0.80	−0.06	0.78
	**GAPU101**	14	8.17	2.33	0.83	0.88	0.05	0.87
	**EMO90**	12	4.41	1.84	0.75	0.77	0.04	0.75
	**EMOL**	9	2.64	1.32	0.46	0.62	0.26	0.58
	**TOTAL**	143						
	**Mean**	13	5.68	1.96	0.71	0.79	0.10	0.77
Whole collection (236)	**DCA03**	12	6.27	2.05	0.83	0.84	0.01	0.824
	**DCA05**	12	5.12	1.93	0.75	0.8	0.07	0.785
	**DCA09**	21	13.61	2.73	0.84	0.93	0.1	0.922
	**DCA13**	15	4.19	1.83	0.45	0.76	0.4	0.738
	**DCA15**	13	4.8	1,9	0.45	0.79	0.43	0.769
	**DCA17**	22	7	2.33	0.64	0.86	0.25	0.844
	**DCA18**	27	9.67	2.63	0.77	0.9	0.14	0.889
	**GAPU71b**	11	5.84	1.99	0.83	0.83	−0.01	0.81
	**GAPU101**	15	7.33	2.25	0.85	0.86	0.02	0.85
	**EMO90**	15	5.08	1.95	0.61	0.8	0.24	0.782
	**EMOL**	10	1.79	0.93	0.19	0.44	0.57	0.411
	**TOTAL**	172						
	**Mean**	16	6.43	2.05	0.66	0.8	0.2	0.78

Size range (bp); Na, number of observed alleles; Ne, number of effective alleles; I, Shannon's information index; Ho, observed heterozygosity; He, expected heterozygosity; F, fixation index; PIC, polymorphic information content.

Estimation of pairwise relatedness (LRM) revealed gave a coefficient ranging from −0.147 to of 0.5, that corresponds to identical genetic profiles. Full identity was revealed between the samples ‘Ogliarola 2, 5, and 6’, between ‘Peranzana 2’ and ‘Peranzana 3’, and between the samples ‘Donna Francesca’ and ‘San Francesco’. These genotypes showed high similarity also with ‘Colmona’ and ‘Lecllin’ (0.4 < LRM < 0.5), while ‘Dolce di Cassano’ was very similar to ‘Dolce di Sannicandro’ (**Table 3**). Several cases of homonymies were observed regarding, in particular, cultivars ‘Ogliarola’, ‘Cellina’, ‘Racioppa’, and ‘Nolca’ (**Table 4**).

**Table 3:** List of pairwise relatedness based on LRM estimator (Lynch and Ritland, 1999).

Genotypes with LRM = 0.5	
OGLIAROLA2	OGLIAROLA5
OGLIAROLA2	OGLIAROLA6
OGLIAROLA5	OGLIAROLA6
PERANZANA2	PERANZANA3
SAN FRANCESCO	DONNA FRANCESCA
**Genotypes with an 0.5 > LRM > 0.4**	
COLMONA	LCELLIN
COLMONA	SAN FRANCESCO
LCELLIN	SAN FRANCESCO
COLMONA	DONNA FRANCESCA
LCELLIN	DONNA FRANCESCA
DOLCE DI CASSANO	DOLCE DI SANNICANDRO
UACCIDIN 2	MELE
RUMANELLA GRAZIANO INTERNA	ROTONDELLA O ROSCIOLA
CAZZALORA	GROSSA DI SPAGNA

**Table 4:** Cases of homonymies based on LRM estimator (Lynch and Ritland, 1999).

Homonyms LRM < 0.2	
CELLINA 7	CELLINA 1, 2, 3, 4, 6, 5, 8
DRITTA 2	DRITTA 1
MELILL 1	MELILL 2
NOLCA 1	NOLCA 2
OGLIAROLA 01	OGLIAROLA 1, 2, 3, 4, 5, 6, 7,8
OGLIAROLA	OGLIAROLA 2, 3, 4, 5, 6, 7, 8
OGLIAROLA 2	OGLIAROLA 3, 8
OGLIAROLA 3	OGLIAROLA 4, 5, 6, 7
OGLIAROLA 4	OGLIAROLA 8
OGLIAROLA 5	OGLIAROLA 8
PENDOLINO TYPE1	PENDOLINO TYPE2
PEPPERINELLA CHIEUTI 1	PEPPERINELLA CHIEUTI 2
PRIMEMEZZANA 3	PRIMEMEZZANA 4
PROVENZALE	PROVENZALE CHIEUTI
PROVENZALE	PRUVENZALE
PROVENZALE CHIEUTI	PRUVENZALE
PASOLINA	PASOLA OSTUNI
PIZZUTA SEPPUNISI	PIZZUTA
PIZZUTA SEPPUNISI	PIZZUTA ESTERNA GRAZIANO
PIZZUTA	PIZZUTA ESTERNA GRAZIANO
RACIOPPA 1	RACIOPPA 2
RACIOPPA 2	RACIUEPP
RUMANELLA 1	RUMANELLA 2
RUMANELLA 2	RUMANELLA GRAZIANO INT.
ROSCIOLA 1	ROSCIOLA 2
ROSCIOLA SERRA	ROSCIOLA 1
ROSCIOLONE	ROSCIOLA 1
SILETTA NISI	SILLETTA 1, 2
TRIGNA	TRIGNO

**Figure 3:**
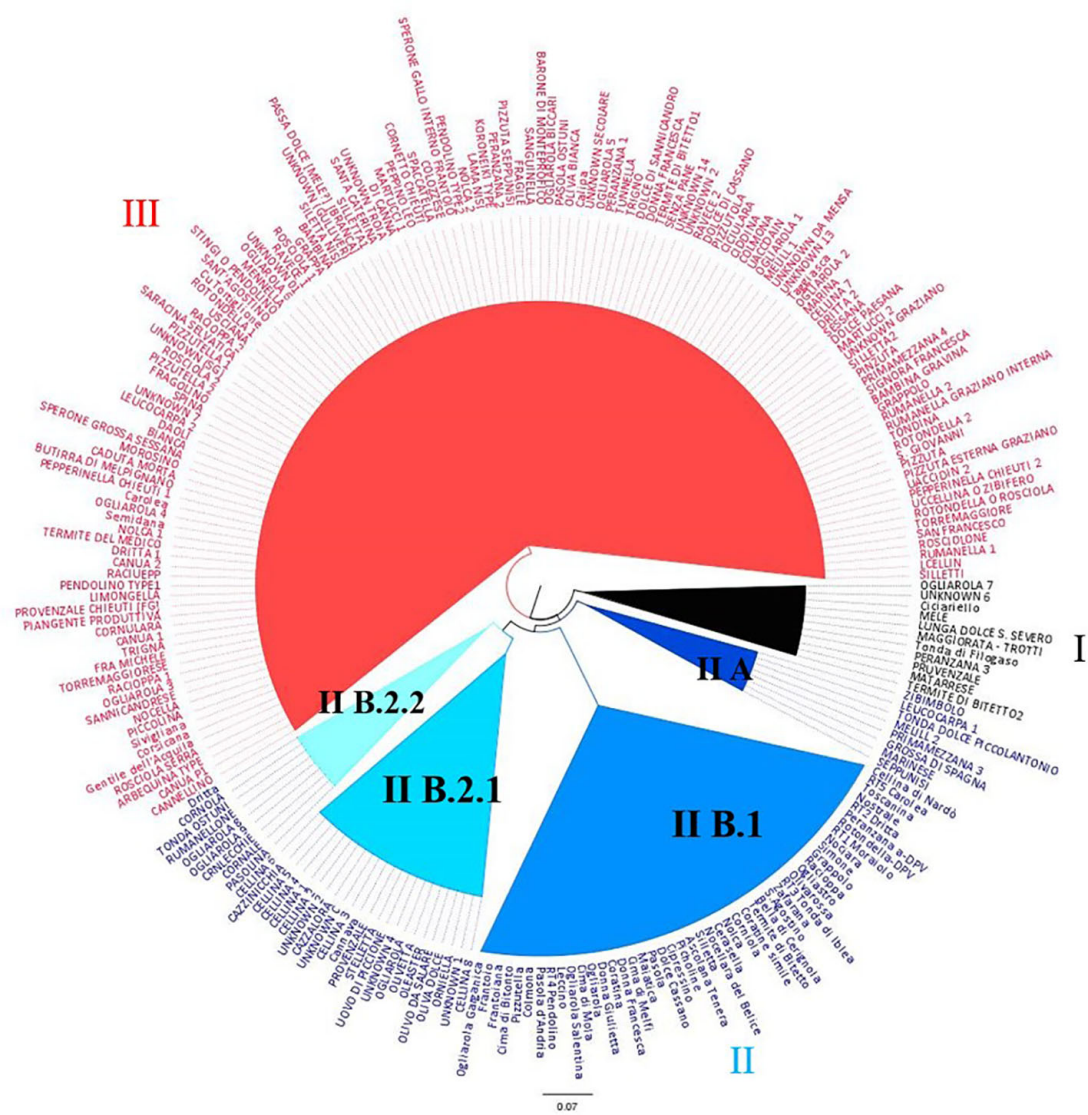
Dendrogram generated by Neighbor-Joining clustering method, illustrating the phylogenetic relationships among 236 olive genotypes, using the SSR markers.

The dendrogram obtained by the Neighbor Joining Analysis disclosed the inter-individual relationship between genotypes, revealing three main clusters (**Figure 3**). Cluster I includes 11 Apulian cultivars and two Calabrian reference cultivars ‘Ciciariello’ and ‘Tonda di Filogaso’. Cluster II includes few table olive varieties (sub-cluster IIA), a group of reference cultivars (sub-cluster II B.1), most of the samples collected in Lecce province, in particular ‘Ogliarola’ and ‘Cellina’ (sub-cluster IIB2.1), and the genotypes characterized by pointed apex and asymmetric stone (‘Crnlecchie’, ‘Cornale’ and ‘Cornola’) (sub-cluster II B2.2). Cluster III includes genotypes coming from all over Apulia except from Lecce province (**Figure 3**).

The population structure indicated a maximum for ΔK at K = 2 (**Supplementary Figure 2**), separating the national Italian germplasm from the local varieties under investigation (**Supplementary Figure 2A**).

### Technological Characterization of Mono-Varietal Oils

FA and sterol composition of the samples under study resulted within the limit set by the European regulations (Commission Regulation (EEC) No 2568/91, Official Journal of the European Communities, 1991) (**Table 5**). Five main fatty acids were detected, with oleic acid being the most abundant, followed by palmitic, linoleic, stearic and palmitoleic. Oleic acid accounted for about 97% of the total Mono Unsaturated Fatty Acids (MUFA) content, ranging from less than 59% (‘Cornale’) to around 80% (‘Bianca’) (mean value of 71.23%). Palmitoleic acid was the second most abundant monounsaturated fatty acid (mean value of 1.52%) ranging from 0.1 (‘Signora Francesca’) to 3.05% (‘Dolce di Cassano’). Among Saturated Fatty Acids (SFA), palmitic acid was the most abundant (85%), ranging from 9.67% (‘Limongella’) to 19.99 (‘Mennella’) (mean value of 14.46%). It was followed by stearic acid (11%) and arachidic acid (0.50%) (**Table 5**). Poly Unsaturated Fatty Acids (PUFA) were the less abundant fatty acids (mean 9.67%), and were represented for more than 96% by linoleic acid (mean value of 9.36%). Linolenic acid showed a mean value of 0.31%, with a minimum of 0.14% (‘Sannicandrese’) and a max. 0.80% (‘Pizzuta Graziano’). Palmitic, oleic and linoleic acids showed the highest variability among the dataset according to the IQR, followed by palmitoleic fatty acid. Oleic/linoleic and MUFA/PUFA ratios were very similar because, as previously reported, oleic and linoleic acids accounted for the great majority of MUFA and PUFA, respectively. Cultivar ‘Bianca’ had the highest value of oleic/linoleic (19.35), while ‘Cornale’ showed the lowest value (3.63), having the lowest and the highest content of oleic and linoleic, respectively. The same genotype showed the highest and lowest values of MUFA/PUFA ratio. About the sterol composition, apparent β-sitosterol was the most abundant form, ranging from 93% (‘Marinese’, ‘Mennella’, ‘Rumanella’ and ‘Silletta’) to 95.3% (‘Torremaggiorese’), highlighting also a small variability among the oils. Campesterol and stigmasterol were the next abundant sterols with mean values of 2.7 and 1.1%, respectively. ‘Racioppa’ featured the lowest amount of campesterol (1.9%) and ‘Sepponisi’ the highest (3.7%) whilst ‘Torremaggiorese’ and ‘Leucocarpa’, and ‘Pizzuta’ cultivars had the minimum and maximum stigmasterol content (0.3 and 2.7%, respectively). Total sterol content was always higher than 1,000 mg kg^−1^ and reached a maximum of 2,791 mg kg in ‘Racioppa’ oil. Fatty acids and sterols of the VOOs were also explored by means of PCA and used to study the similarity among the oils (**Supplementary Figure 3**). The first two principal components (PCs) explained around 35% of the total dataset variability. In particular PC1, which explained 26% of the data variability, was mostly affected by oleic and linoleic acids together with the sum of MUFA, PUFA and the respective ratios, while PC2, which explained about 9.5% of the data variability, was strongly influenced by single saturated fatty acids (C_17:0_, C_20:0_) and by sterols. No clear samples clusters were observed in the score plot and the majority of them stand around the origin.

**Table 5:** Descriptive statistics of the purity characteristics of the monovarietal oils obtained from 34 minor olive Apulian accessions.

	Min	Max	Mean	Q1	Q3	IQR
*Fatty acids composition (area %)*						
Myristic acid C_14:0_	0.00	0.03	0.01	0.00	0.02	0.02
Palmitic acid C_16:0_	9.67	19.99	14.46	12.52	16.08	3.56
Palmitoleic acid C_16:1_	0.10	3.05	1.52	0.83	2.08	1.25
Margaric acid C_17:0_	0.00	0.31	0.09	0.04	0.13	0.09
Heptadecenoic acid C_17:1_	0.02	0.33	0.12	0.08	0.14	0.06
Stearic acid C_18:0_	0.97	2.82	1.91	1.65	2.16	0.51
Oleic acid C_18:1_	58.73	79.73	71.23	69.16	74.36	5.20
Linoleic acid C_18:2_	4.12	16.18	9.36	7.61	10.70	3.09
Linolenic acid C_18:3_	0.14	0.80	0.31	0.23	0.36	0.13
Arachidic acid C_20:0_	0.22	0.60	0.50	0.46	0.56	0.10
Gadoleic acid C_20:1_	0.15	0.49	0.34	0.30	0.39	0.10
Behenic acid C_22:0_	0.00	0.18	0.08	0.05	0.11	0.06
Lignoceric acid C_24:0_	0.00	0.20	0.03	0.00	0.05	0.05
SFA	12.63	22.39	17.07	15.59	18.33	2.74
MUFA	62.29	81.27	73.21	71.67	75.98	4.31
PUFA	4.51	16.41	9.67	7.92	11.09	3.18
Oleic/Linoleic	3.63	19.35	8.47	6.53	9.64	3.10
MUFA/PUFA	3.80	18.02	8.33	6.47	9.48	3.01
*Sterols composition (area %)*						
Cholesterol	0.00	0.40	0.20	0.00	0.20	0.20
Brassicasterol	0.00	0.10	0.00	0.00	0.00	0.00
Campesterol	1.90	3.70	2.70	2.40	3.00	0.60
Campestanol	0.10	1.00	0.40	0.30	0.50	0.30
Stigmasterol	0.30	2.70	1.10	0.80	1.40	0.60
Δ-7-Campesterol	0.00	0.90	0.20	0.00	0.30	0.30
Apparent β-Sitosterol	93.00	95.30	93.50	93.10	93.70	0.60
Δ-7-Stigmastenol	0.00	0.50	0.30	0.20	0.30	0.10
Δ-7-Avenasterol	0.10	0.80	0.40	0.30	0.60	0.30
Total sterols (mg kg)	1017	2791	1846	1390	2222	832

Q1, first quartile; Q3, third quartile; IQR, inter-quartile range; SFA, total saturated fatty acids; MUFA, total mono-unsaturated fatty acids; PUFA, total poly-unsaturated fatty acids.

### Evaluation of Phytosanitary Status and Sanitation

Out of 129 analyzed genotypes, only 16 satisfied the sanitary status “virus free”. While no infection associated to the viruses ArMV, SLRV, OLV-1, OLV-2, CMV, TNV and to the bacterium *X. fastidiosa* was found on all the samples, 106 genotypes resulted infected by OLYaV, two genotypes by CLRV and five genotypes by both OLYaV and CLRV (**Supplementary Table 7**). Forty-four genotypes infected by OLYaV and CLRV were submitted to sanitation treatments, and 30 and 14 genotypes were treated respectively, with *in vivo* and *in vitro* thermotherapy, followed by micrografting (**Supplementary Table 7**). Thirty-eight out of 44 genotypes were assessed free from OLYaV and CLRV, 25 by *in vivo* thermotherapy and 13 by *in vitro* thermotherapy. Overall, 81 “healthy plants” were produced, including the 76 coming from sanitation treatments. Moreover 42 out of them, belonging to 20 presumed different varieties, also genetically and pomologically characterized, were maintained in screen house as Primary Sources to be shortly registered in the national certification system (DDG 06/12/2016).

### Database Release and Olive Germplasm Management

All data obtained from the molecular, morphological, phytosanitary and technological characterization were collected in a database integrated with the regional GIS portal (**Supplementary Figure 4**), accessible on request to Apulia region website (misura 10.2.1: www.psr.regione.puglia.it). The database contains additional information about the GPS position of field collections, reports of public meetings, consulted documents related to olive cultivation history, and geotagged photographs related to the recovered genotypes, the pictures of the countryside and rural landscape where the genotypes were found and further detailed pictures about leaves, flowers, fruits and stem.

## Discussion

Olive tree is a primary economic source for Apulia region, where it has a wide and ancient varietal richness, still largely uncharacterized and not exploited. In order to enhance the production of oil and table olives, it is essential to proceed to an accurate varietal identification and characterization of this germplasm. The regional Re.Ger.O.P. project allowed to identify, collect and characterize this marginalized germplasm investigating morphological, molecular, technological and phytosanitary aspects, and supported *in situ* and *ex situ* conservation. Based on the bibliographic sources, notary studies, meetings, archives and libraries, numerous varieties locally cultivated in the past centuries, were identified, mainly in the minor olive groves, or as single specimens' relicts of old orchards. A total of 177 olive genotypes were collected from all the Apulian provinces and compared with a set of 59 Italian cultivars already characterized and used as reference. Although several authors have already described the genetic diversity of ancient and local Italian cultivars and evaluated the phenological, physiological and molecular characteristics (Erre et al., 2012; Lombardo et al., 2019), this work represents the first investigation conducted with a multidisciplinary approach.

The morphological characterization of 97 minor genotypes based on descriptors of leaf, fruit, and stone, revealed an extremely variability within the Apulian olive germplasm. Forty-nine genotypes were recognized as already described varieties, while 64 were found to be known only with local name or nameless. These genotypes could be both the result of the processes of hybridization or between cultivars or between cultivars and wild oleaster naturally present in Apulian countryside. The dendrogram obtained using the morphological markers separated the germplasm in three main clusters. One group included the white-fruit genotypes, such as ‘Oliva bianca’ and Leucocarpa, and genotypes characterized by the pointed apex and asymmetric stone, such as ‘Cornale’, ‘Cornulara’ etc. A second group included various phenotypes, while the third group included 29 genotypes characterized by medium-high weight and size of fruit and stone, including several important table olives such as ‘Grossa di Spagna’, ‘Sant'Agostino’, ‘Rosciolone’, ‘Grappolo’ and ‘San Francesco’, and the most typical Apulian ‘sweet olives’, such as ‘Dolce di Cassano’, and ‘Dolce di Sannicandro’. This clusterisation was not supported by the SSRs analysis since the microsatellites did not separate the cultivars according to the fruit size and weight. In contrast, this clustering was obtained by Montemurro et al. (2005) using AFLPs and by D'Agostino et al. (2018) using GBS analysis, probably due to the multilocus strategy of these two approaches.

Both the Neighbor Joining dendrogram and Structure analysis revealed a clear differentiation between the national Italian germplasm from the local one, indicating that it could have a different origin. The richness in alleles of the Apulian germplasm is also confirmed by the high total number of alleles in Cluster II that includes the large part of the Apulian genotypes. It is interesting to observe that in the Cluster II, that grouped both Apulian genotypes and national cultivars, the total number of alleles is higher than Cluster III. This could be explained by the fact that the national cultivars originated by different ancestors coming from different parts of Mediterranean basin (D'Agostino et al., 2018); thus, they are characterized by a more heterogeneous gene pool reflecting their multiple origin.

The genetic analysis confirmed a large genetic diversity on the whole sample of the olive trees analyzed, indicating an observed heterozygosity lower than the expected heterozygosity. This is in contrast with the fact that olive is a predominantly allogamous species (Diaz et al., 2006; Farinelli et al., 2008), but similar results were obtained by other researches on Apulian olive germplasm (Muzzalupo et al., 2009; Boucheffa et al., 2017; di Rienzo et al., 2018b). This could be explained with the fact that reproduction in olive is strongly influenced by the rating of self-incompatibility, which is, in turn, under the effects of the genetic control but also environmental and climatic conditions (Lavee, 2013; Montemurro et al., 2019).

The Lynch and Ritland analysis highlighted only a single true case of synonymy between the genotype ‘Donna Francesca’ and ‘San Francesco’, probably as the result of a misnaming. On the contrary, several homonymies were observed, mostly regarding the varieties ‘Ogliarola’ and ‘Cellina’, that are the two most common cultivars in southern Apulia area. It is probable that, under the generic denomination ‘Ogliarola’ (meaning ‘producer of oil’), different genotypes derived by clonal variation or spontaneous crossing between cultivars and/or feral forms, are included (Muzzalupo et al., 2014; D'Agostino et al., 2018). ‘Ogliarola’ and ‘Cellina’ are both susceptible to *X. fastidiosa* (Giampetruzzi et al., 2016) but the showed great variability among samples of these two cultivars could represent an interesting aspect in order to recognize a different behaviour of the plants to the disease.

Morphological and molecular analyses clustered together the genotypes ‘Peranzana’, ‘Provenzale’, ‘Dritta’ ‘Torremaggiorese’ and ‘Torremaggiore’. These varieties all originate from the Sub-Appennino Dauno area, which is a geographical area characterized by mountains that create the conditions for the genetic isolation of olive genotypes. It is possible that these varieties have a common genetic background to those introduced in Apulia from the south France (Provenza) at the end of 1700 (Fiore, 2018).

Oil macro and micro components defines the product's nutritional, qualitative and sensorial features (Boskou, 2006; D'Imperio et al., 2007; Rotondi et al., 2010). Fatty acids and sterols profiles and content greatly affect the nutritional and stability characteristics of the product and they are strongly linked to the genotype.

Overall, the VOOs obtained from the 34 studied Apulian accessions were generally rich in MUFA content, which was always higher than 60% of total fatty acids, and oleic acid appears to be the most significant variable affecting the samples distribution. Oleic acid influences oil stability to oxidation (Choe and Min, 2009) and plays an important nutritional role (Huang and Sumpio, 2008). The samples ‘Cornale’, ‘Mennella’, ‘Oleaster’, ‘Racioppa’ and ‘Sannicandrese’ showed the lowest amount of oleic acid (below 70%) and were well separated from the cloud in the PCA score plot. About linoleic acid content, in 28 out of 34 samples it ranged from 7.33 to 16.18%, values quite higher than those reported for others Apulian typical virgin oils such as Coratina (Aparacio and Luna, 2002; Rotondi et al., 2010). In particular, ‘Nolca’, ‘Cornale’, ‘Fragolina’, ‘Mennella’, ‘Oleaster’, ‘Pasola’, ‘Pizzutella’, ‘Racioppa’, ‘Sig. Francesca’, ‘Sannicandrese’, ‘Silletta Nisia’, ‘Silletta’, ‘Torre Maggiorese’ and ‘Pasolina’ were characterized by a linoleic content higher than 10%.

Although several variables could affect the oxidative stability of the oils, in particular the antioxidants content (Velasco and Dobarganes, 2002), it is well known that oleic/linoleic and MUFA/PUFA ratios are useful indices for forecasting VOO stability. Modification in the contents of oleic and linoleic acids are related to different factors, including the latitude and altitude of olive cultivation (Inglese et al., 2011). A minimum ratio oleic/linoleic of 7 was established as an indicator of oil oxidative stability (Kiritsakis et al., 1998). In our samples, this ratio was highly variable, with 23 oils showing the minimum ratio oleic/linoleic >7, thus forecasting a good stability and shelf-life of VOOs. In particular, oil of cultivar Bianca stands out for a very high ratio oleic/linoleic, thus it is expected to be very stable to oxidation.

About the sterols total content, it is well known that sterols contribute to the oil antioxidant activity (Choe and Min, 2009) and have positive effect on human health (Kritchevsky and Chen, 2005; Regulation (EU) No 432/2012). A wide variability among the VOOs was observed, ranging between 1,017 mg kg^−1^, close to the minimum EU limit (Commission Regulation (EEC) No 2568/91), and 2,791 mg kg^−1^, which is quite higher than values generally reported for VOOs (Manai-Djebali et al., 2012; Lukić et al., 2013). ‘Racioppa’ oil, in particular, showed the highest total sterols content and could be interesting as a source for breeding programs and commercial valorization.

The high linolenic content is typically present in naturally sweet olives, and it might be an indicator for increased desaturase activity for the conversion of oleic acid to linoleic acid (Aktas et al., 2014). The virgin olive oil from ‘Dolce di Cassano’ which is a “naturally debittered olive” used for both oil extraction and for cooked consumption, presents the highest levels of palmitoleic, margaric and linolenic acids. The sweetness of this cultivar is an interesting character that could be exploited in order set up a pilot trial for the production of frozen olives ready to be cooked in fry pan.

In conclusion, this research has shown the presence of genotypes with interesting technological features that deserve to be deeper explored. The conservation of these genetic resources should be implemented by measures that minimize the risk of spreading diseases such as *Xylella*. The phytosanitary analyses showed that Apulian olive germplasm is in overall good phytosanitary status. OLYaV was the predominant viral agent, in accordance with the past evidences in Southern Italy (Fontana et al., 2019), while only seven genotypes were infected by CLRV. The sanitation treatments adopted resulted very effective in eliminating both viruses with 83% and 92% efficiency for *in vivo* and *in vitro* thermotherapy, respectively. Thus, the protocols here described, resulted to be very efficient and could be suggested to simultaneously and quickly remove these two viruses. The 42 primary sources produced by Re.Ger.O.P project have been officially declared compliant to the phytosanitary requirements for the commercialization of certified materials in EU (Annex I of DDG 6/12/2016).

Several works have been made on the phenotypic and genetic variability of local olive germplasm at regional level (Erre et al., 2010; Hmmam et al., 2018; Mousavi et al., 2019; Rotondi et al., 2018) including Apulia region (Salimonti et al., 2013; di Rienzo et al., 2018a). Nevertheless, this study represents the first investigation conducted with a multidisciplinary approach, taking into consideration several aspects about the biodiversity of the Apulian germplasm. The Re.Ger.O.P. project has allowed, through the integration of different activities and competences, to bring out, in the Apulian olive germplasm, a richness, certainly not unexpected, but now completely ascertained. The cultural history of this region, thanks to the geographical location that has make it a transit point for centuries, explains the complexity of relationships existing among the hundreds of cultivars of the wide Apulian olive-growing panorama. At the same time, we can observe how, in Apulia, a heritage of unique genetic diversity has been preserved, which certainly deserve to be deepened and valued. The information obtained are encouraging, and they will help to valorize this germplasm with economic advantages and it will prevent genetic erosion.

## Data Availability Statement

This article contains previously unpublished data. The name of the repository and accession number(s) are not available.

## Author Contributions

VF, FB, SC, CM, FC, and GB designed the experiment. PV, GV, AP, and GA collected the plant material. GB, VM, AS, and GL performed the sanitary state characterization. FC, GS, and GD performed the technological characterization and analyses. SC and GV performed the morphological characterization and analyses. VR, MM, CM, SS, IM, WS, and GM performed the molecular characterization and analyses. IM, CM, FC, MM, and SC were involved in data interpretation. IM, VR, MM, FC, SC, CM, and GL wrote the manuscript. MM, IM, and CM implemented the manuscript. All authors read and approved the final manuscript.

## Funding

This research was supported by Apulia region within the: PROGRAMMA SVILUPPO RURALE FEASR 2014–2020 Asse II “Miglioramento dell'Ambiente e dello Spazio Rurale” Misura 10.2.1 “Progetti per la conservazione e valorizzazione delle risorse genetiche in agricoltura”-trascinamento della Misura 214 Az. 4 sub azione a) del PSR 2007–2013 Progetti integrati per la biodiversità—Progetto Re.Ger.O.P. “Recupero del Germoplasma Olivicolo Pugliese” Progetto di continuità.

## Conflict of Interest

Authors MC, LG, BG, FV and FB are participants to SINAGRI s.r.l. without any kind of salary; VR, IM, WS, and AP were consultants of SINAGRI s.r.l.

The remaining authors declare that the research was conducted in the absence of any commercial or financial relationships that could be construed as a potential conflict of interest.
