# Supplementary figures and images for: Re.Ger.O.P.: An Integrated Project for the Recovery of Ancient and Rare Olive Germplasm
Source: Front Plant Sci. 2020 Feb 20;11:73. doi: 10.3389/fpls.2020.00073 (PMC7044272; doi:10.3389/fpls.2020.00073)

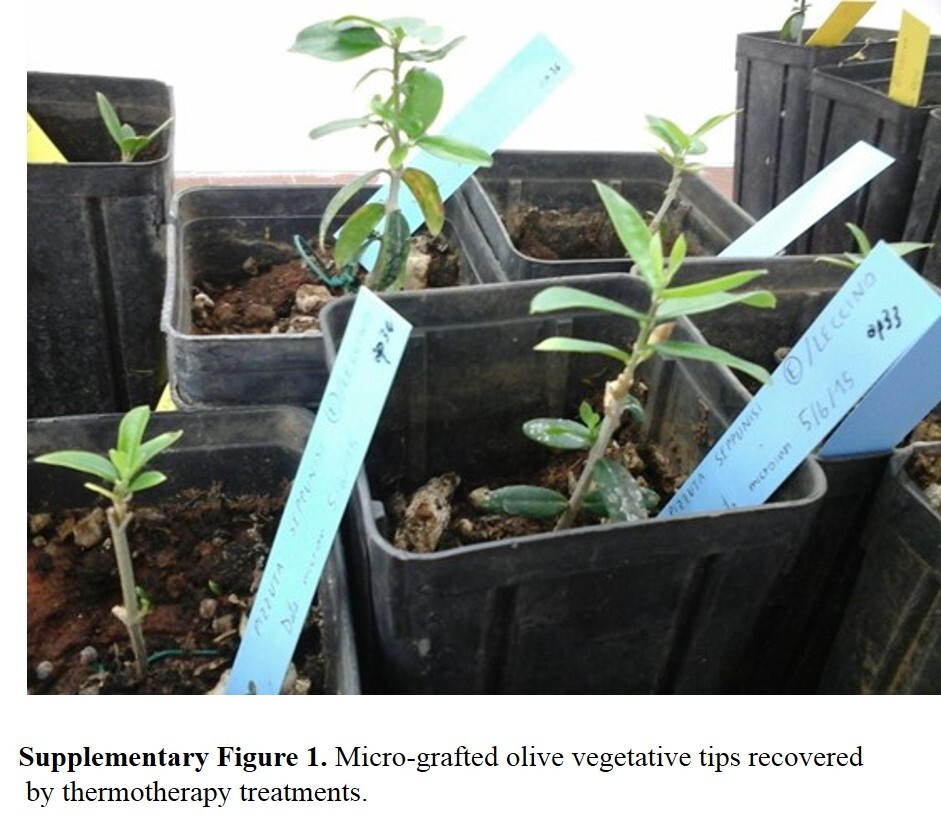

Supplement: Supplementary file 2 [file Image_1.jpeg]

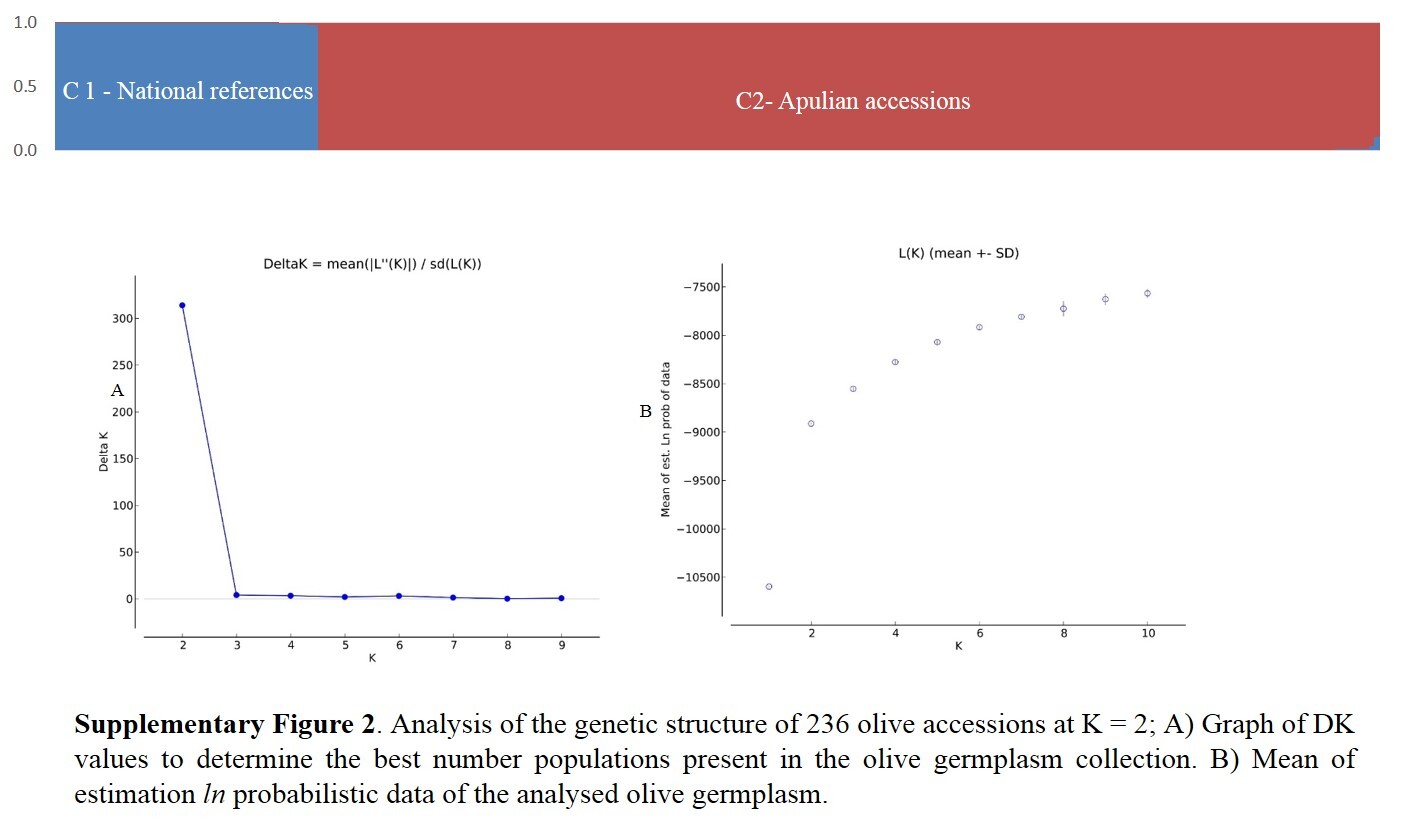

Supplement: Supplementary file 3 [file Image_2.jpeg]

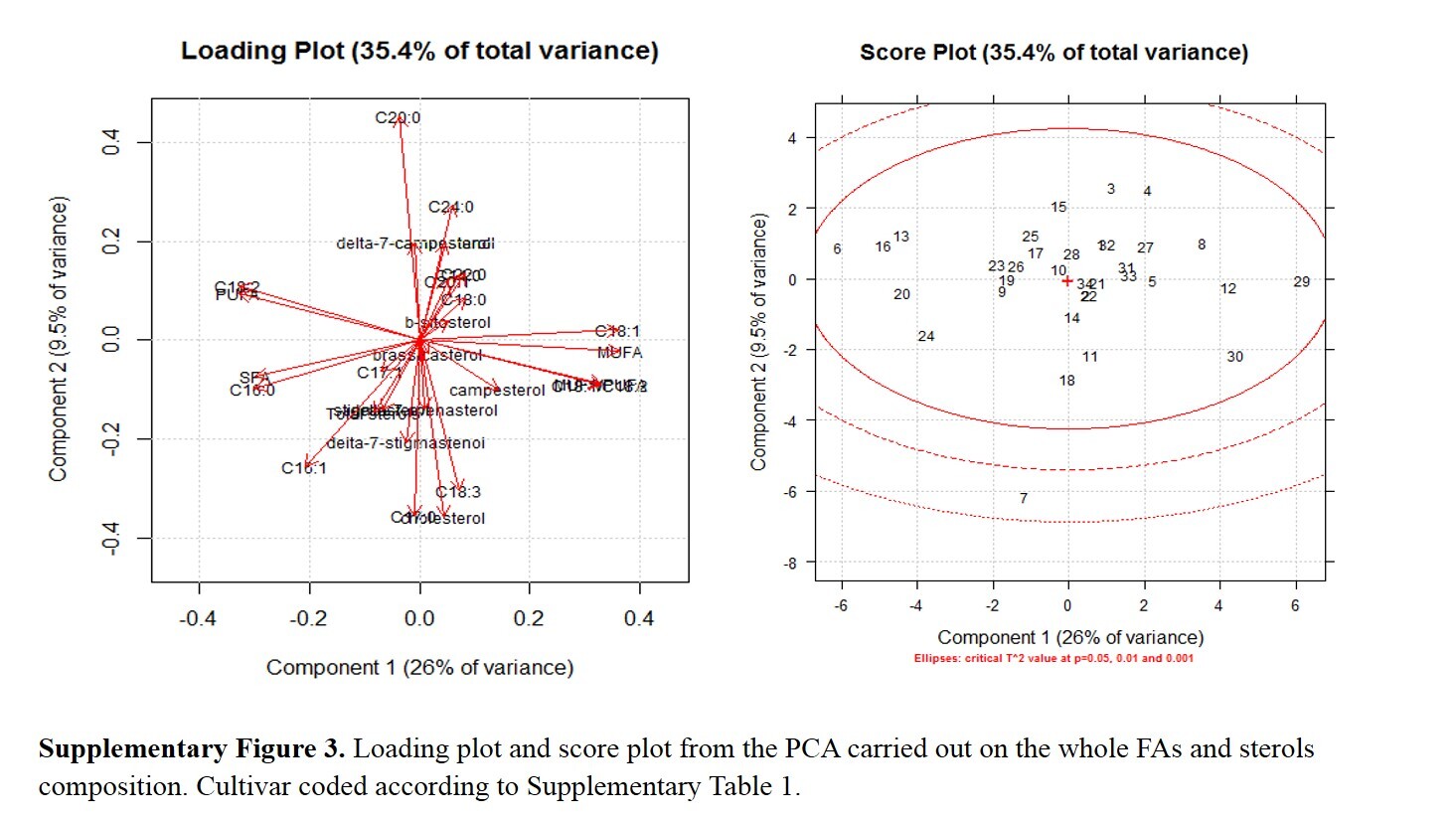

Supplement: Supplementary file 4 [file Image_3.jpeg]

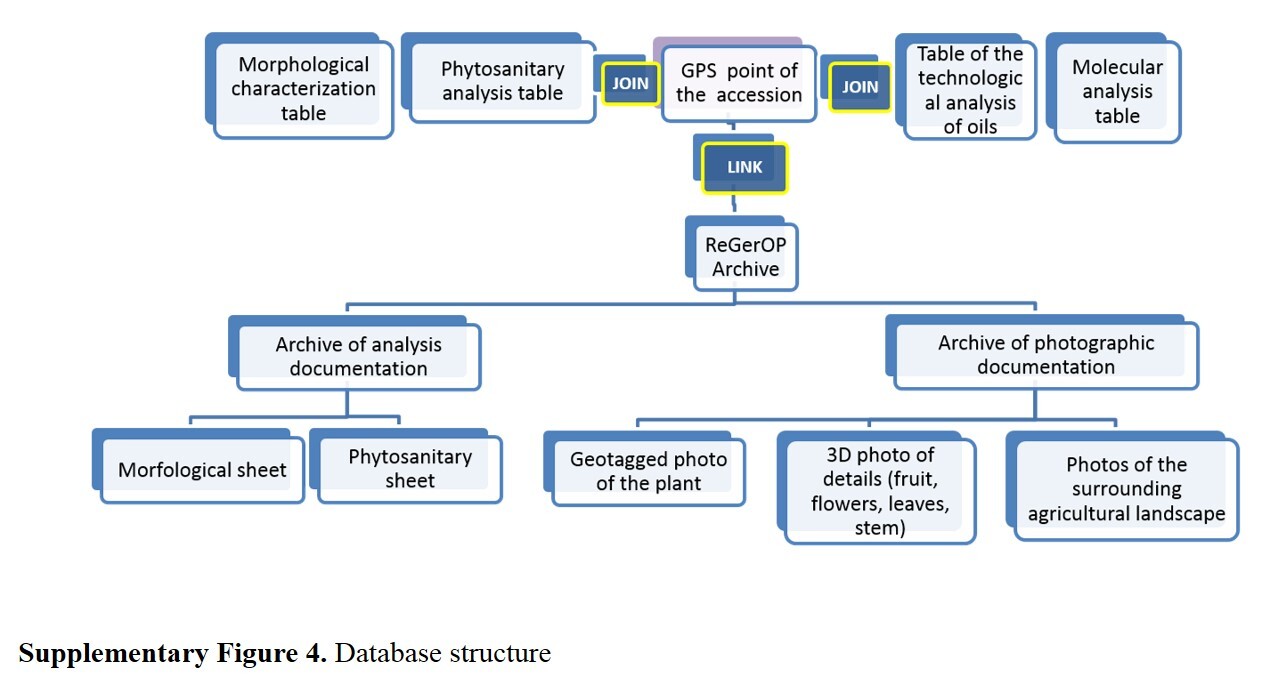

Supplement: Supplementary file 5 [file Image_4.jpeg]
